# Isolation, characterization, and functional verification of salt stress response genes of NAC transcription factors in *Ipomoea pes-caprae*

**DOI:** 10.3389/fpls.2023.1119282

**Published:** 2023-02-01

**Authors:** Yiren Su, Yang Liu, Shizhuo Xiao, Yuan Wang, Yitong Deng, Lukuan Zhao, Yao Wang, Donglan Zhao, Xibin Dai, Zhilin Zhou, Qinghe Cao

**Affiliations:** Jiangsu Xuzhou Sweetpotato Research Center/Sweetpotato Research Institute, Chinese Academy of Agricultural Sciences, Xuzhou, China

**Keywords:** *I*. *pes-caprae*, NAC transcription factor, abiotic stress, expression analysis, salt-tolerance, sweetpotato

## Abstract

Adverse environmental stress is a major environmental factor threatening food security, which is why improving plant stress resistance is essential for agricultural productivity and environmental sustainability. The NAC (NAM, ATAF, and CUC) transcription factors (TFs) play a dominant role in plant responses to abiotic and biotic stresses, but they have been poorly studied in *Ipomoea pes-caprae*. In this research, 12 NAC TFs, named IpNAC1–IpNAC12, were selected from transcriptome data. The homologous evolution tree divided IpNACs into four major categories, and six *IpNACs* were linearly associated with *Arabidopsis ANAC* genes. From the gene structures, protein domains, and promoter upstream regulatory elements, IpNACs were shown to contain complete NAC-specific subdomains (A–E) and cis-acting elements corresponding to different stress stimuli. We measured the expression levels of the 12 *IpNACs* under abiotic stress (salt, heat, and drought) and hormone treatment (abscisic acid, methyl jasmonate, and salicylic acid), and their transcription levels differed. IpNAC5/8/10/12 were located in the nucleus through subcellular localization, and the overexpressing transgenic *Arabidopsis* plants showed high tolerance to salt stress. The cellular Na^+^ homeostasis content in the mature and elongation zones of the four *IpNAC* transgenic sweetpotato roots showed an obvious efflux phenomenon. These conclusions demonstrate that *IpNAC5/8/10/12* actively respond to abiotic stress, have significant roles in improving plant salt tolerance, and are important salt tolerance candidate genes in *I*. *pes-caprae* and sweetpotato. This study laid the foundation for further studies on the function of *IpNACs* in response to abiotic stress. It provides options for improving the stress resistance of sweetpotato using gene introgression from *I*. *pes-caprae*.

## Introduction

*Ipomoea pes-caprae* (Linn.) R. Br. (*Convolvulaceae* 2n = 2x = 30) mainly grows on tropical and subtropical beaches (Miryeganeh et al., 2003), and it has anti-inflammatory (Cristiane et al., 2017) and antitumor effects (Manigauha et al., 2015), relieves gout (Akinniyi et al., 2022), and has significant curative effects. Compared with other crops, it has obvious biological characteristics, such as salt tolerance (Liu et al., 2020), as well as drought (Suarez, 2011) and high temperature resistance (Cheng et al., 2021). It is an important medicinal and coastal sand fixation greening plant (Akinniyi et al, 2022). In the plant evolutionary process, variation in the natural environment is one of the most important factors affecting plant growth and development (Hawrot-Paw et al., 2016). To cope with abiotic stress (drought, salt, and high temperature), plants have formed a variety of complex and precise biological resistance mechanisms (Li et al., 2021). In many stress signaling pathways, transcription factors (TFs), as multi-functional proteins, play a crucial role in plant defense mechanisms by controlling gene expression and regulating the basic functions of plants (Falak et al., 2021; Hrmova and Hussain, 2021).

NAC (NAM, ATAF, and CUC) proteins comprise a unique TF family in plants (Wang et al., 2021). It has five highly conserved subdomains A–E (Meng et al., 2018). Domains C and D are responsible for binding to DNA (Du et al., 2020), domain A is involved in the formation of functional dimers (Nakashima et al., 2012), and domains B and E determine the functional diversity of NAC genes (Puranik et al., 2012). With the development of biotechnology, NAC TFs in different species have been identified, including 117 in *Arabidopsis thaliana* (Ooka et al., 2003), 151 in *Oryza sativa* (Nuruzzaman et al., 2010), and 152 in *Nicotiana tabacum* (Rushton et al., 2008). As one of the largest transcription families in plants, NAC TFs have many functions, such as in plant secondary metabolism (Srivastava and Sahoo, 2021), plant development and senescence (Gong et al., 2020), and hormone regulation (Tao et al., 2022). In response to high salt, drought, and high temperatures, NAC TFs play an important regulatory function by activating or inhibiting the expression of target genes and improving abiotic stress tolerance (Trishla and Kirti, 2021; Guo et al., 2022). Ju et al. (2020) overexpressed *VvNAC17* in *Arabidopsis*, enhancing tolerance to salt, osmotic, and freezing stress and increasing ABA sensitivity. Li et al. (2021) suggested that *GmNAC06* could cause the accumulation of proline and glycine betaine alleviate the effects of reactive oxygen species (ROS), and maintain the homeostasis of the Na*^+^* ion. Therefore, *GmNAC06* overexpression in hairy roots led to significant salt tolerance in the whole composite plant. Duan et al. (2017) verified that *MfNACs* combine with the *MtGlyl* promoter to improve plant drought tolerance by regulating glutathione back to the reduction state.

Although the NAC family has been widely studied, there have been few reports about *I*. *pes-caprae* and its related species. Many genes in wild relatives provide genetic resources for germplasm improvement (Zhang et al., 2016). *Ipomoea pes-caprae*, as a wild relative of sweetpotato, can be used as a model plant for studying the salt tolerance mechanism of sweetpotato. China has abundant sweetpotato resources, and its planting area and yield rank first in the world (Kim et al., 2018). However, the increasing occurrence of extreme temperatures, drought, and severe secondary salinization seriously limits sweetpotato growth, development, and yield. Sweetpotato is a salt-sensitive plant (Meng et al., 2020), and there has been little research on its tolerance to salt. It is necessary to utilize related gene resources in *I*. *pes-caprae* to improve the cultivated sweetpotato. Therefore, screening wild relatives for salt-tolerance genes to study salt tolerance mechanisms is particularly important to breed new sweetpotato varieties adapted to salt stress and other abiotic stressors. In our study, according to the transcriptome data, 12 *I*. *pes-caprae IpNAC* genes encoding NAC proteins were screened and identified. Phylogenetic relationship, gene structure, and protein domain analyses were carried out. Genetic expression profiles under different abiotic stressors and hormone treatments were studied. IpNAC5, IpNAC8, IpNAC10 and IpNAC12 were localized in the nuclei. Through heterologous overexpression in *Arabidopsis*, the plant phenotype in 180 mM NaCl treatment was observed, and Na^+^ flow in sweetpotato roots in mature and elongation zones was measured under salt stress. *IpNAC5/8/10/12* were preliminarily verified to participate in resistance to abiotic stress, such as salt stress. This study laid a foundation for utilizing *I*. *pes-caprae* genes to improve sweetpotato stress resistance.

## Materials and methods

### Plant materials

The experimental materials—*I*. *pes-caprae*, ‘ZiShu8’, *Nicotiana tabacum*, and *Arabidopsis thaliana*—were cultivated at the Xuzhou SweetPotato Research Center, China. *Nicotiana* and *Arabidopsis* were grown at 20–28 °C under a 16 h/8 h dark/light cycle with 50–60% humidity and 500 µmol·m^−2^·s^−1^ light intensity. *Ipomoea pes-caprae* was cultivated in Hoagland nutrient solution. After 3–5 weeks of growth, 7–9 functional leaves and 8–12 cm roots were present, and *I*. *pes-caprae* plants were transferred to soil mixed with fine sand and a nutritional substance (3:1). Then, after 4–5 weeks, we selected plants with similar growth characteristics for experiments.

### Isolation of salt stress-responsive *I*. *pes-caprae IpNAC* genes

The amount of expressed genes was obtained from transcriptome data (Liu et al., 2020). Combined with the level of *I*. *pes-caprae* NAC differentially expressed genes under salt stress, 12 TFs were preliminarily screened as research targets and named *IpNAC1–12*. The *IpNAC* nucleotide sequence was converted into an amino acid sequence by NCBI^1^. The physicochemical properties of the IpNAC1–12 proteins, including the theoretical molecular weight (MW), isoelectric point (pI), and hydrophobicity, were analyzed using the Expasy ProtParam tool^2^. The full-length amino acid sequences of the IpNAC and ANAC proteins were used to generate a phylogenetic tree by deleting gaps and blanks. The unrooted neighbor-joining tree was constructed after ClustalW alignment using MEGA (Tamura et al., 2011). Bootstrap analysis was performed with 100 replicates to assess the level of statistical support for each tree node (Hu et al., 2015). EvoView was used for subsequent beautification.

### Gene structure, protein domain, and collinearity analyses of *IpNAC* genes

The MEME^3^ online tool was used to identify the protein domains of the IpNAC, with the following parameters: site distribution = zero or one occurrence per sequence, largest number of motifs = 10, and optimum motif width of 6–50. Default settings were used for the other parameters. The results were presented by GSDS 2.0^4^. The gene sequences of *IpNACs* were compared with the full-length sequences by SnapGene 4.36 to determine the exon–intron structure and were visualized using TBtools software. Collinearity between *I*. *pes-caprae (IpNACs)* and *A*. *thaliana* (TAIR)^5^ (*ANACs*) was determined using MCScanX (Wang et al., 2013), and the figures were generated using Circos (Darzentas, 2010).

### Cis-element analysis of promoter sequences

Most gene regulatory regions are located 2 kb upstream of the translation start site. The *IpNAC* promoter sequences were obtained from the *I*. *pes-caprae* genome database (unpublished). Then, the PlantCARE^6^ online database was used to identify the cis-acting elements of these promoters.

### Plant hormone and abiotic stress treatments

We selected plants with similar growth rates for plant hormone and abiotic stress treatments. Untreated plants were used as controls. The leaves or adventitious roots were collected after 0, 3, 6, 12, 24, or 48 h of treatment. Heat treatment was performed by transferring *I. pes-caprae* to a 45 °C climate box, and the roots were submerged in 20% PEG 6000 for 48 h for dehydration treatment. The leaves and adventitious roots were harvested from the plants. The roots of *I. pes-caprae* were submerged in a salt solution at three concentrations of 300, 600, and 900 mM NaCl, and adventitious roots were harvested. For hormone treatment, adventitious roots were submerged in 0.1 mM abscisic acid (ABA), 2 mM methyI jasmonate (MeJA), or 2 mM salicylic acid (SA) for 0, 3, 6, 12, 24, or 48 h, and the adventitious roots were collected. The roots were collected from three separate plants at each time point of each treatment and combined to form one sample. All experiments were performed independently and in triplicate. The collected samples were immediately frozen in liquid nitrogen and stored at −80 °C.

### RNA isolation and quantitative real-time PCR

Following the manufacturer’s instructions, the FastPure Plant Total RNA Isolation Kit (Vazyme, China) was used to isolate the total RNA in the leaves and roots. The quality of the RNA was determined using a NanoDrop2000c spectrophotometer (Thermo Fisher Scientific, US), and total RNA integrity was identified by electrophoresis on 1.0% agarose gel. The HiScript II 1st Strand cDNA Synthesis kit (Vazyme, China) was used to obtain cDNA from total RNA. Real-time quantitative PCR was carried out using SYBR green (TOYOBO, Japan) on an ABI QuantStudio 6 Flex (Thermo Fisher Scientific, US) with a final volume of 10 µL per reaction. Each reaction mixture contained 5 µL SYBR Green Realtime PCR Master Mix (TOYOBO, Japan), 0.4 µL forward primer (10 μM), 0.4 µL reverse primer (10 μM), 3.2 µL ddH_2_O, and 1 µL cDNA template (diluted at 1:10). Specific primers for each gene are listed in **Supplementary Table S3**. The cycling parameters were 95 °C for 1 min and 40 cycles of 95 °C for 15 s, 60 °C for 15 s, and 72 °C for 45 s. The *Actin* gene of *I*. *pes-caprae* was used as an internal control. Each measurement was performed with three biological replicates. Data were analyzed using the 2^-ΔΔCt^ method.

### Construction of overexpression vectors

The coding DNA sequences of *IpNAC1–12* were obtained from the transcriptome datasets of *I*. *pes-caprae*. We designed the specific primers (**Supplementary Table S2**). The Phanta Max Super-Fidelity DNA Polymerase (Vazyme, China) (18 μL ddH_2_O; 25 μL 2 × Phanta Max Buffer; dNTP Mix (10 mM each); forward primer (10 μM); reverse primer (10 μM); 1 μL Phanta Max Super-Fidelity DNA Polymerase; 1 μL cDNA) were used to amplify the *IpNAC1–12* genes from the cDNA of *I*. *pes-caprae*. The PCR parameters were set as follows: initial denaturation at 95 °C for 3 min, 30 cycles of denaturation at 95 °C for 15 s, annealing at 58 °C for 30 s, extension at 72 °C for 1 min 30 s, and final extension at 72 °C for 10 min. The PCR products were subcloned into the pHB-GFP vector with a *Hin*d III restriction site to form pHB-IpNAC1-12-GFP. pHB-IpNAC1-12-GFP and pHB (set as a control) were transformed into *Arabidopsis*. In addition, using pCAMBIA0390-DsRed as the backbone vector, the coding regions of *IpNAC1–12* were inserted into the pCAMBIA0390-DsRed expression vector constructing pUBI.U4:: IpNAC1-12-CaMV35S::DsRed for *Agrobacterium rhizogenes-mediated* transformation. pCAMBIA0390-DsRed was used as a control. Each experiment was conducted using more than three biological replicates.

### Subcellular localization of IpNAC5, IpNAC8, IpNAC10, and IpNAC12 in tobacco

The 35S: GFP-IpNAC5/IpNAC8/IpNAC10/IpNAC12 plasmid were transformed into *Agrobacterium tumefaciens* strain GV3101 for plant transformation. Transient expression in tobacco leaves was determined according to a published method (Zhou et al., 2018). After transformation, tobacco was kept in the light room for 36 h before examination by fluorescence microscopy. Then, the tobacco leaf epidermis was peeled to make a temporary squash. After incubation with phosphate-buffered saline containing 40, 60-diamidino-2-phenylin dole (DAPI), the temporary squashes were observed under a Leica Fluorescence Microscope at 10 × 20 (Leica Microsystems, Wetzlar, GmbH). The image data were processed using Adobe Photoshop (Mountain View, CA, USA). All transient expression assays were repeated at least three times.

### Salt stress treatment of transgenic *Arabidopsis*

The recombinant vectors pHB-IpNAC5/IpNAC8/IpNAC10/IpNAC12-GFP were transferred into *Arabidopsis* through the *Agrobacterium tumefaciens* strain GV3101 using the floral dipping method. After transformation, the T3 (third generation) seeds were germinated on 1/2 MS medium with 50 mg L^−1^ hygromycin for screening. Then, the WT and overexpressing transgenic seeds were cultivated on 1/2 MS medium with 180 mM NaCl at 22 °C under 16 h of daylight. After 10 d, the primary root length was measured and the growth state was observed.

### *Agrobacterium rhizogenes*-mediated transformation

The pUBI.U4:: IpNAC5/IpNAC8/IpNAC10/IpNAC12-CaMV35S::DsRed constructs were introduced into *A*. *rhizogenes* strain K599. ZiShu 8 with the same growth state was selected for *Agrobacterium* infection according to the published method (Yu et al., 2020). Subsequently, the transformed plants were transferred into the soil under a high-humidity (28 °C/65%) environment. After three weeks, the positive plants were selected through the portable fluorescent lamp instrument, immersed in hydroponic solution for 3 d, and then collected.

### Cellular Na^+^ homeostasis flux measurements

The WT and *IpNAC5/8/10/12* transgenic sweetpotato were cultured in 150 mM NaCl solution for 24 h. First, 3–4 cm of intact root tips were collected and fixed in a buffer solution for 30 min. Then, Na*^+^* flux in the root elongation zone was recorded for 5 min. The root mature zone was measured for another 5 min. Each group had five replicates.

### Statistical analysis

The data are presented as the mean ± standard deviation of the mean (SD), and statistically significant differences were assessed using Dunnett’s test at *p < 0.05* as the point of minimal statistical significance in all analyses.

## Results

### Identification and analysis of salt stress-responsive *IpNAC* genes

**Figure 1:**
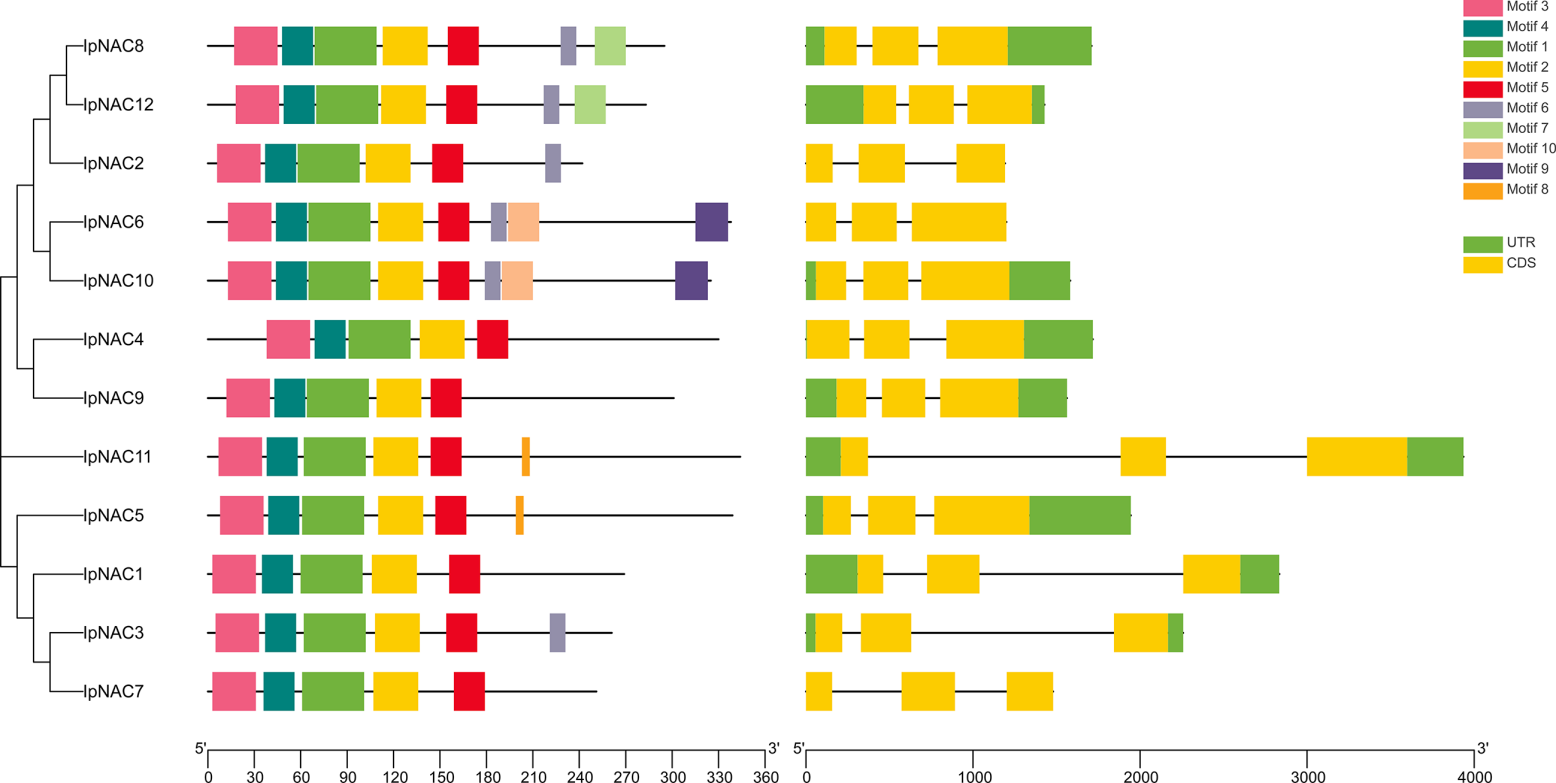
Analysis of the conserved motifs and gene structures of *Ipomoea pes-caprae IpNAC* genes. The conservative motif distribution and homologous tree of IpNACs are located on the left side, which is expressed by a color box. The black scale lines represent the relative protein length. The gene structure is on the right side, the exon and intron are represented by a yellow rectangle and black line, respectively, and the untranslated region (UTR) is represented by a green rectangle. Gene size can be expressed by the scale.

We identified 12 NAC genes in *I*. *pes-caprae*, named *IpNAC1–12*. We found that *IpNAC1–12* had high homology with *Arabidopsis*, sweetpotato, and their wild species. *IpNAC2* is one of the reported genes, and *NAC1* was screened from the cDNA library of *I*. *pes-caprae*, but its function was not clear. We verified that *IpNACs* contained a complete ORF. Analysis of the physicochemical properties revealed amino acid lengths of IpNACs ranging from 242 to 344. The molecular weight varied from 27.58 to 39.21 KDa, and the pI ranged from 5.24 to 8.52. Except for IpNAC2, IpNAC3, and IpNAC6, IpNAC proteins were weakly acidic and were predicted to be located in the nucleus (**Supplementary Table S1**). IpNAC proteins contain NAC family characteristic subdomains (A–E) (**Figure 1**; **Supplementary Figure S1**).

### Syntenic relations, phylogenetic analysis, and classification of *IpNAC* and *ANAC* genes

**Figure 2:**
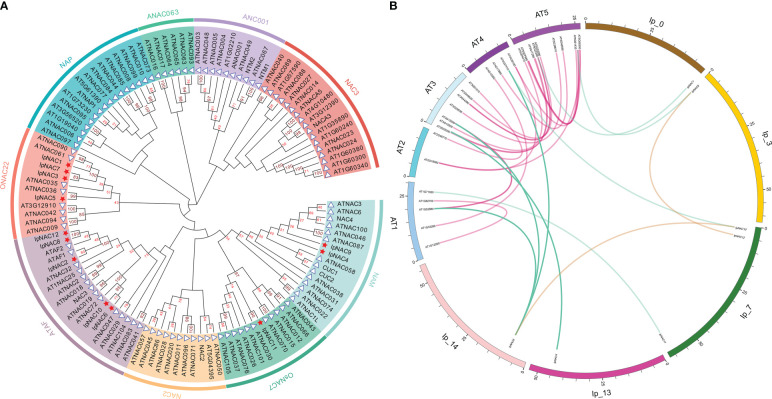
Comparative analysis of *Ipomoea pes-caprae IpNACs* and *Arabidopsis ANAC* genes. **(A)** Phylogenetic tree of IpNAC and ANAC proteins. The full-length amino acid sequences of the IpNAC proteins were used to construct the phylogenetic tree with MEGA X through 100 bootstrap tests and the Poisson model method. The number at the node indicates the bootstrap value. The nine subfamilies, namely ONAC22, NAP, ANAC063, ANAC001, NAC3, NAM, OsNAC7, NAC2, and ATAF, were set in different colors as the background. IpNACs are represented by red pentagons, and ANACs are represented by blue triangles. (AtNAC=ANAC). **(B)** Collinearity analysis of NAC genes in *I pes-caprae* and *Arabidopsis*. The chromosomes of *I pes-caprae* and *Arabidopsis* are expressed in different colors, and the positions of *IpNAC* and *ANAC* genes in chromosomes are marked. The purple curve represents the collinear region of *ANAC* genes, the brown curve represents the collinear region of *IpNAC* genes, and the blue curve represents the collinear region of *ANACs* and *IpNACs*.

To investigate the taxonomic and phylogenetic relationships of IpNACs, we used the model plant *Arabidopsis* to analyze the potential function of the IpNAC protein. Using MEGA-X software, we performed phylogenetic analysis of the IpNAC and ANAC proteins (without duplicated and erroneous sequences) to establish an unrooted phylogenetic tree (**Figure 2A**). The NAC proteins were classified into nine subfamilies. IpNACs were divided into ONAC22 (including IpNAC1, IpNAC3, IpNAC5, and IpNAC7), ATAF (including IpNAC2, IpNAC6, IpNAC8, IpNAC10, and IpNAC12), OsNAC7 (including IpNAC11), and NAM (including IpNAC4 and IpNAC9). To obtain more information about *IpNAC* gene*s*, we identified syntenic relationships between *IpNACs* and *ANACs* and found six pairs of syntenic *NAC* genes between *I*. *pes-caprae* and *Arabidopsis* (**Figure 2B**). Interestingly, one *IpNAC* gene (*IpNAC6*) and one *ANAC* gene (*ANAC072*) were associated with three or two syntenic blocks, respectively. At the same time, other *IpNAC* genes have a close relationship with *ANAC* genes, indicating that their functions may be similar.

### Motif distribution, gene structures, and regulatory elements of *IpNAC* genes

**Figure 3:**
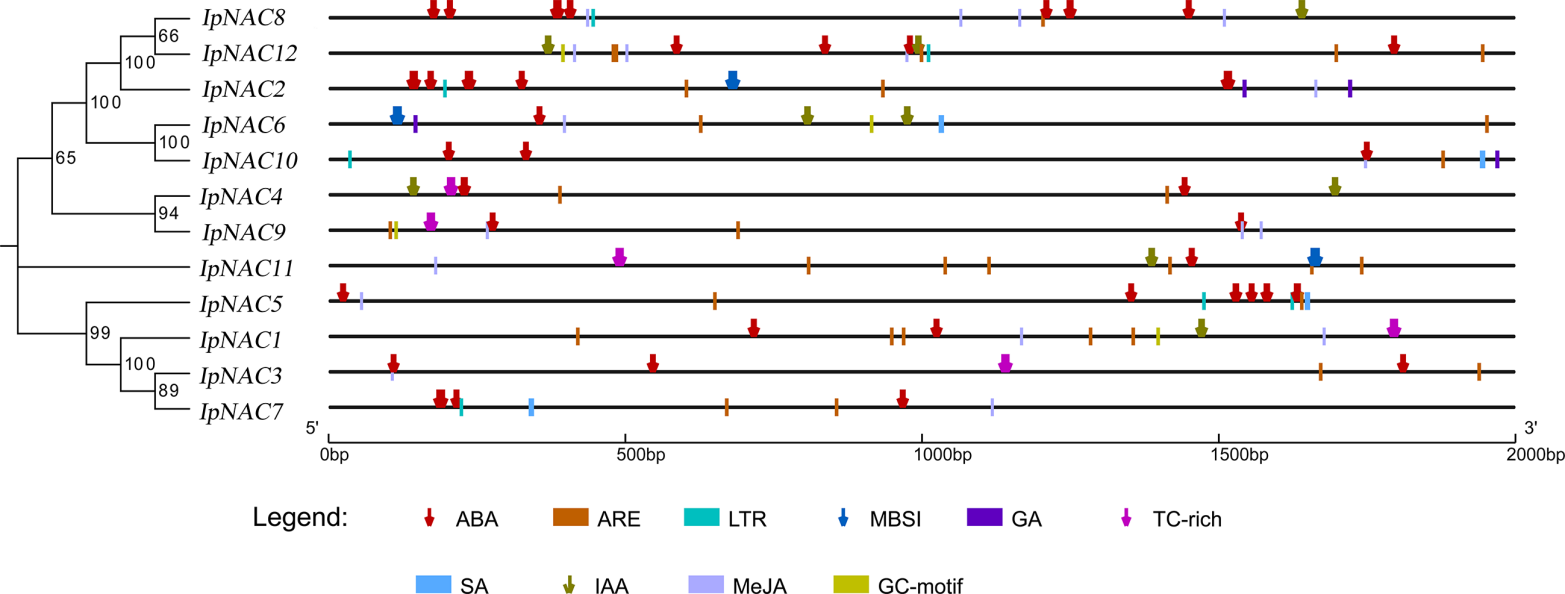
Cis-elements in the 2 kb upstream promoter of *Ipomoea pes-caprae IpNACs*. The homologous tree of the *IpNAC* genes is located on the left, and the number at the node indicates the bootstrap value. Symbols of different colors and shapes mark the relative positions of different elements, and the black scale line represents the relative protein length.

Motifs are key functional units. The IpNAC motif components were analyzed using MEME online software. All IpNAC proteins contained the NAC family characteristic subdomains (A–E), named motif1/2/3/4/5, located at positions 0–210. Except for IpNAC1/4/7/9, other protein sequences had more than five motifs, with a maximum of eight. The 12 *IpNACs* had three exons, separated by two introns (**Figure 1**), forming a highly conserved gene structure. The untranslated region (UTR) lengths differed significantly, indicating a difference in the efficiency of *IpNAC* gene expression. TFs regulate gene function by binding cis-acting elements to the promoter. The PLACE Online website was used to analyze the 2 kb upstream region of the transcription start site of *IpNACs*, and a series of stress response elements were found (**Figure 3**). A cis-acting element involved in drought-inducibility (MBIS) was found in three *IpNAC* genes, an enhancer-like element involved in anoxic specific inducibility cis-acting element (GC-motif) was detected in four *IpNAC* genes, and a defense and stress response cis-acting element (TC-rich) was detected in five *IpNAC* genes. Anaerobic induction cis-acting regulatory elements (AREs) were identified in 11 *IpNAC* genes. Interestingly, there were five *IpNAC* genes with low-temperature responsive cis-acting elements (LTRs). Many hormone-responsive elements were found in the *IpNAC* promoter sequence. GA responsive elements were found in three *IpNAC* genes. SA responsive elements were found in four *IpNAC* genes. IAA responsive elements were found in 11 *IpNAC* genes, and ABA responsive elements were found in 12 *IpNAC* genes. This indicates that plant hormones play a central role in the regulation of the salt stress response.

### *IpNAC* genes expression under salt stress

**Figure 4:**
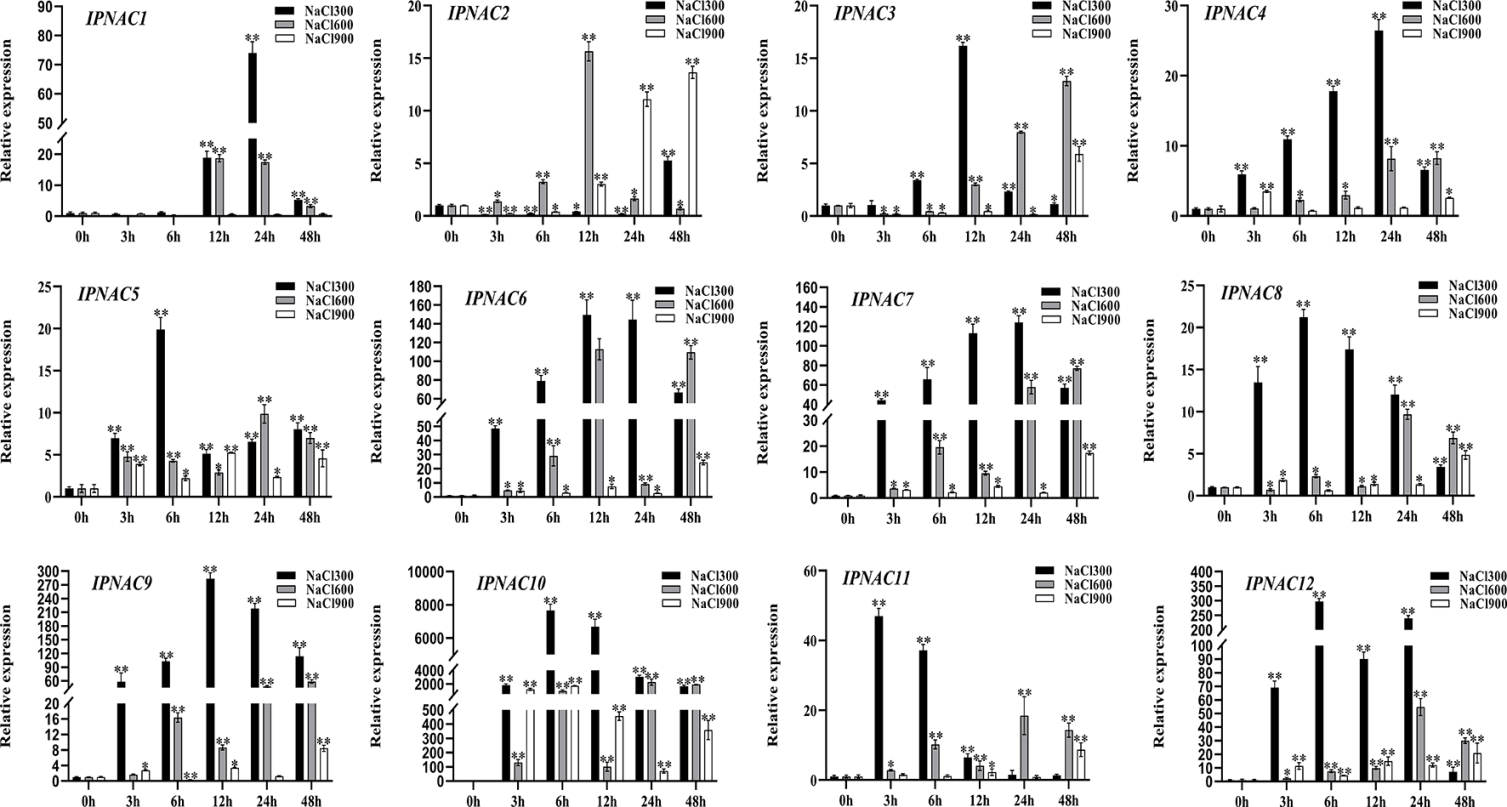
The expression level of *IpNAC* genes under different salt treatments. The roots were soaked in 300, 600, and 900 mM NaCl solutions and collected at different time points (0, 3, 6, 12, 24, and 48 h). The expression of *IpNAC* genes under salt treatment was detected by quantitative real-time PCR. Non-stressed plants (0 h) were used as a control, and each experiment was conducted with three biological replicates and three technical replicates. The mean values and SD were obtained from the experiment. The *p-values* were evaluated using Student’s *t*-test. Asterisks indicate the level of significance, **p < 0.01 and *0.01 < p < 0.05. Transcript levels at 0 h were set as 1.

*Ipomoea pes-caprae* is a highly salt-tolerant crop, and the expression pattern of *IpNACs* was determined after treatment with different concentrations of NaCl solution. Among the 12 genes, there were significant differences (**Figure 4**). Compared with 600 and 900 mM NaCl solutions, *IpNAC* genes treated with 300 mM NaCl solution had the highest response to salt induction. Interestingly, *IpNAC10* showed the strongest significance after different salt solution treatments and maintained a high mRNA level (>2000-fold) at 6 and 24 h treatment periods. *IpNAC11* was upregulated significantly, reaching the maximum change (47-fold) at 3 h, and returned to baseline over time. In contrast, *IpNAC2* reached maximum expression at 48 h (6-fold), and the trend was downregulated and only slightly upregulated throughout the treatment period. *IpNAC5*, *IpNAC8*, and *IpNAC12* expression were induced 19–300-fold at 6 h. The expressions of *IpNAC3*, *IpNAC6*, and *IpNAC9* were increased 15–300-fold at 12 h, and those of *IpNAC4* and *IpNAC7* expression reached 25–130-fold at 24 h. Under 600 mM NaCl treatment, the 12 genes were not strongly induced at 3 and 6 h. *IpNAC1* and *IpNAC2* were upregulated after 12 h (16–19-fold) and remained stable or were downregulated in the early stage. The induction level of *IpNAC5*, *IpNAC*8, *IpNAC11*, and *IpNAC12* increased 10- to 60-fold (24 h) and were upregulated steadily throughout the period. The expressions of *IpNAC3*, *IpNAC4*, *IpNAC6*, *IpNAC7*, and *IpNAC9* reached 15- to 280-fold (48 h). Interestingly, *IpNAC6*, *IpNAC7*, and *IpNAC9* were upregulated, but the trend was high–low–high. The *IpNAC* genes were generally downregulated after 900 mM NaCl solution treatment, and *IpNAC2*, *IpNAC3*, *IpNAC4*, *IpNAC6*, *IpNAC7*, *IpNAC8*, *IpNAC9*, *IpNAC11*, and *IpNAC12* reached the maximum significance level at 48 h, among which *IpNAC2*, *IpNAC3*, and *IpNAC4* were downregulated (3–24 h), and *IpNAC5* and *IpNAC10* maintained a high induction at 6 h. Interestingly, *IpNAC1* did not respond to 900 mM NaCl solution treatment.

### *IpNAC* genes expression under heat and drought stress

**Figure 5:**
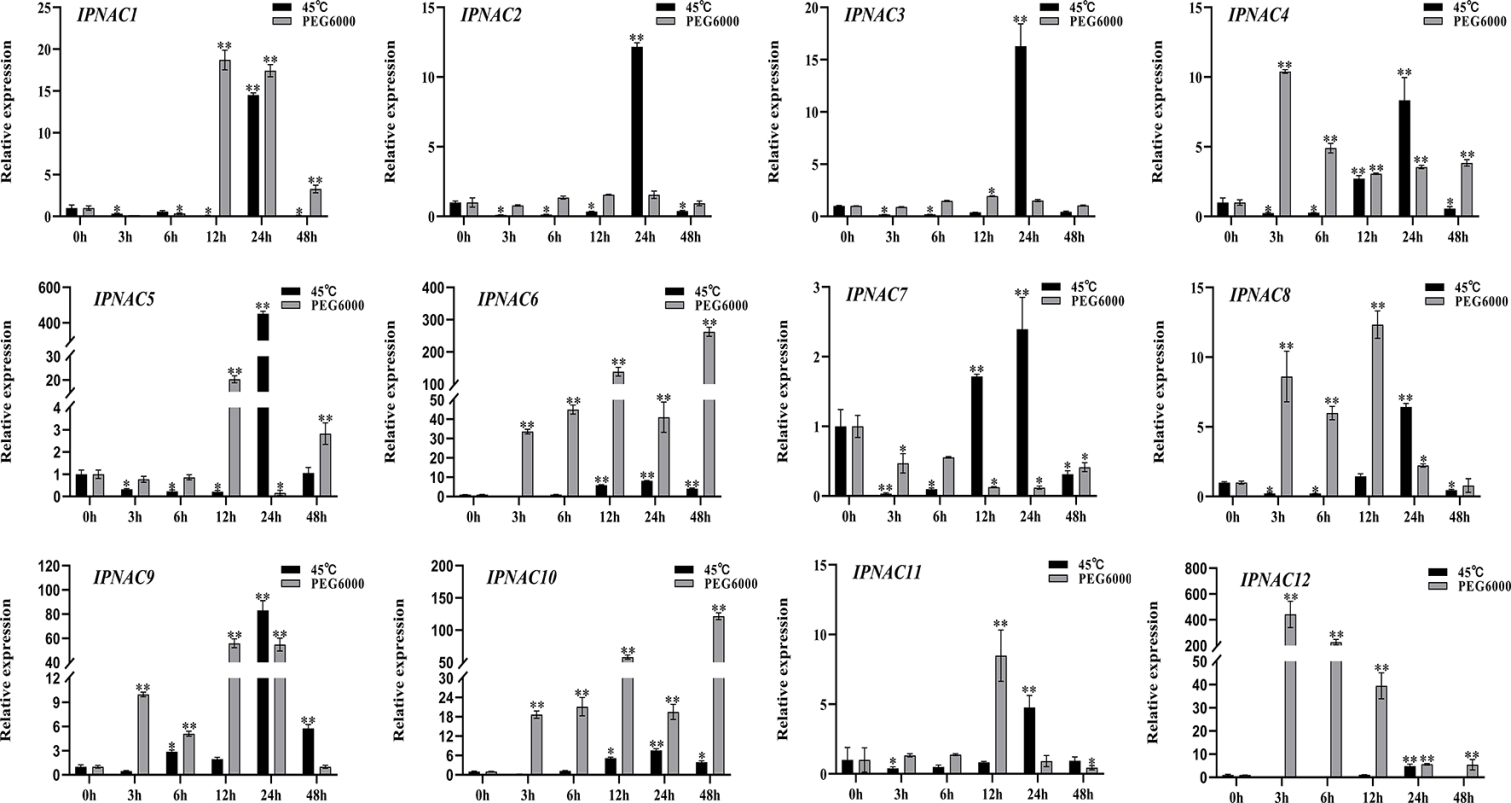
The expression level of *IpNAC* genes under heat (45 °C) and 20% PEG 6000 solution stress treatments. The roots were soaked in 20% PEG 6000 solutions and collected at different time points (0, 3, 6, 12, 24, and 48 h). Non-stressed plants (0 h) were used as a control, and each experiment was conducted with three biological replicates and three technical replicates. The mean values and SD were obtained from the experiment. The *p-values* were evaluated using Student’s *t*-test. Asterisks indicate the level of significance, **p < 0.01 and *0.01 < p < 0.05. Transcript levels at 0 h were set as 1.

*Ipomoea pes-caprae* is a high temperature- and drought-tolerant crop. The expression level of most *IpNAC* genes was increased under heat stress (45 °C) and drought stress (20% PEG 6000), but the change in the expression level under drought stress was more obvious than that under high temperature stress (**Figure 5**). Under a high temperature (45 °C), the *IpNAC5* induction level was the highest (451-fold), followed by that of *IpNAC1*, *IpNAC2*, *IpNAC3*, and *IpNAC9*, which reached the highest significant expression (10–90-fold) at 24 h. However, the maximum induction of *IpNAC4*, *IpNAC6*, *IpNAC7*, *IpNAC8*, *IpNAC9*, *IpNAC10*, *IpNAC11*, and *IpNAC12* was less than 10-fold. Interestingly, all *IpNAC* genes were strongly induced at 24 h under heat stress. Most *IpNAC* genes were near or below the baseline at 3–24 h and near the baseline at 48 h. Under drought stress, *IpNAC12* reached a maximum induction at 48 h (442-fold), followed by *IpNAC1*, *IpNAC2*, *IpNAC3*, *IpNAC5*, *IpNAC8*, *IpNAC9*, and *IpNAC11*, reaching a maximum at 12 h (1–80-fold). *IpNAC4*, *IpNAC6*, and *IpNAC10* increased 10- to 300-fold at 3 and 48 h. However, the expression trends of *IpNAC2* and *IpNAC3* after high temperature and drought stress were consistent (**Figure 5**). In addition, compared with high temperature stress, *IpNAC2* and *IpNAC3* were less responsive to drought stress. Under the same stress, *IpNAC6* and *IpNAC10* showed similar trends but were less responsive to high temperature stress.

### *IpNAC* genes expression under different hormone stressors

**Figure 6:**
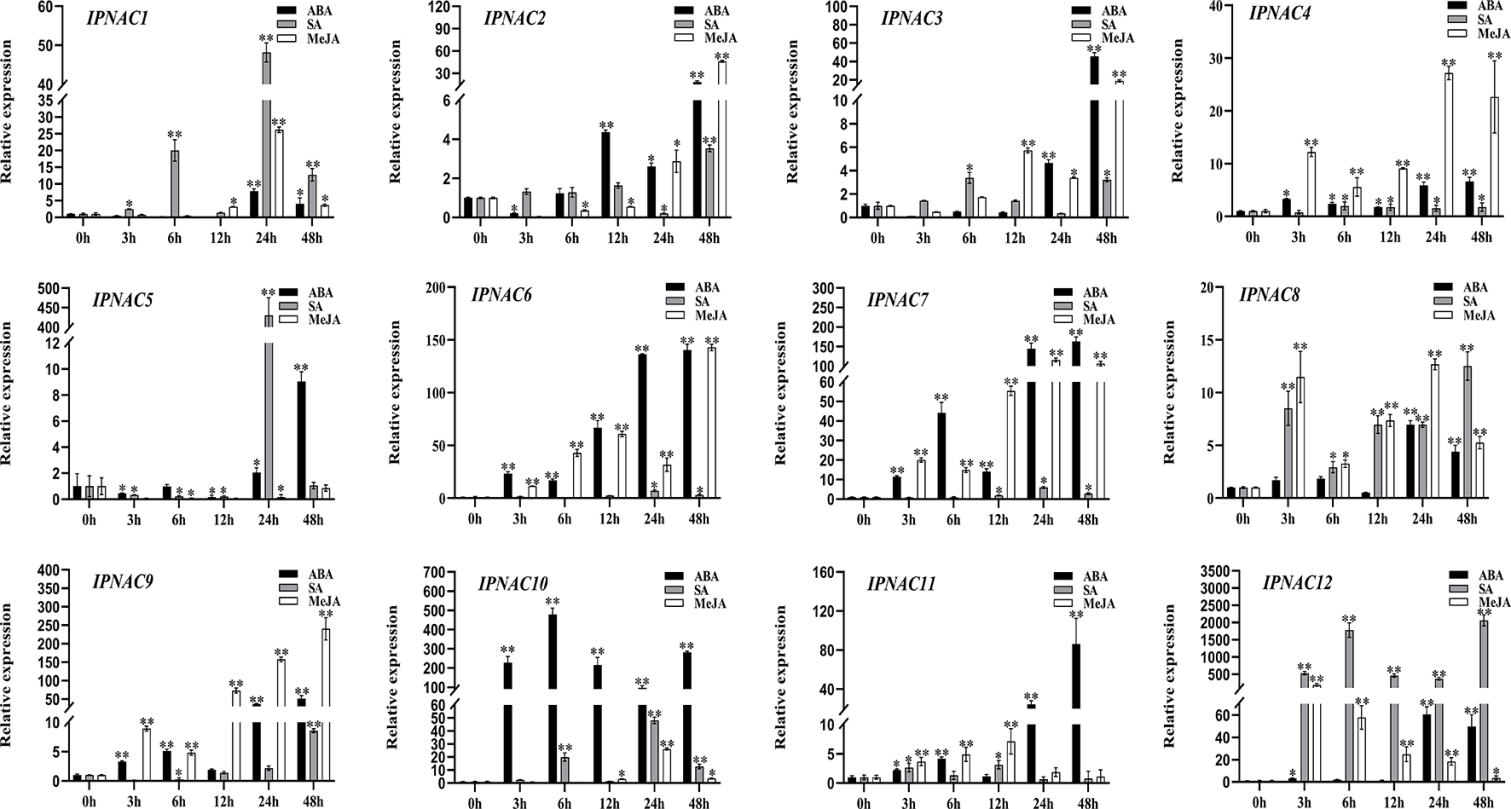
The expression level of *IpNAC* genes in the plant hormone treatment. The roots were soaked in 0.1 mM abscisic acid (ABA), 2 mM salicylic acid (SA) solution, or 2 mM jasmonic acid (JA) solution, and the roots were collected at different time points. Non-stressed plants (0 h) were used as a control, and each experiment was conducted with three biological replicates and three technical replicates. The mean values and standard deviation (SD) were obtained from the experiment. The *p-values* were evaluated using Student’s *t*-test. Asterisks indicate the level of significance, **p < 0.01 and *0.01 < p < 0.05. Transcript levels at 0 h were set as 1.

In response to internal and external stress, plant hormones (abscisic acid [ABA], salicylic acid [SA], and methyl jasmonate [MeJA]) play an important role in the stress signal network. We studied the relationship between the transcriptional levels of the 12 *IpNAC* genes under different plant hormones (**Figure 6**). Gene expression of *IpNAC4*, *IpNAC6*, *IpNAC7*, *IpNAC9*, *IpNAC10*, *IpNAC11*, and *IpNAC12* after ABA treatment increased throughout the treatment period, and most genes were rapidly upregulated 5- to 500-fold at 24 and 48 h, while *IpNAC10* quickly reached 477-fold at 6 h. Similarly, the induction levels of *IpNAC1*, *IpNAC2*, *IpNAC3*, *IpNAC5*, and *IpNAC*8 reached maximum significance (4–60-fold) at 12–24 h, and the early response was weak or downregulated. Under SA stress, *IpNAC1*, *IpNAC8*, and *IpNAC12* were significantly upregulated throughout the treatment period. The induced expression of *IpNAC12* reached 2064-fold at 48 h. *IpNAC2*, *IpNAC3*, *IpNAC4*, *IpNAC5*, *IpNAC6*, *IpNAC7*, *IpNAC9*, *IpNAC10*, and *IpNAC11* were mainly upregulated (2–500-fold) at 6–24 h, and except for the highest induction time, the induction times were close to baseline or downregulated. Based on the results of the MeJA treatment, the transcript level of *IpNAC9* reached a peak of 241-fold at 48 h. *IpNAC4*, *IpNAC6*, *IpNAC7*, *IpNAC8*, *IpNAC11*, and *IpNAC12* also maintained a high level (5–150-fold) throughout the period. Most trends continuously increased from 3 to 24 h and steadily decreased from 24 to 48 h. In contrast, *IpNAC1*, *IpNAC2*, *IpNAC3*, *IpNAC5*, and *IpNAC10* were downregulated (2–60-fold) in different periods (3–12 h). At the same time, *IpNAC5* did not respond to MeJA induction and was not sensitive throughout the period (**Figure 6**).

### IpNAC5/8/10/12 localized in the nuclei

**Figure 7:**
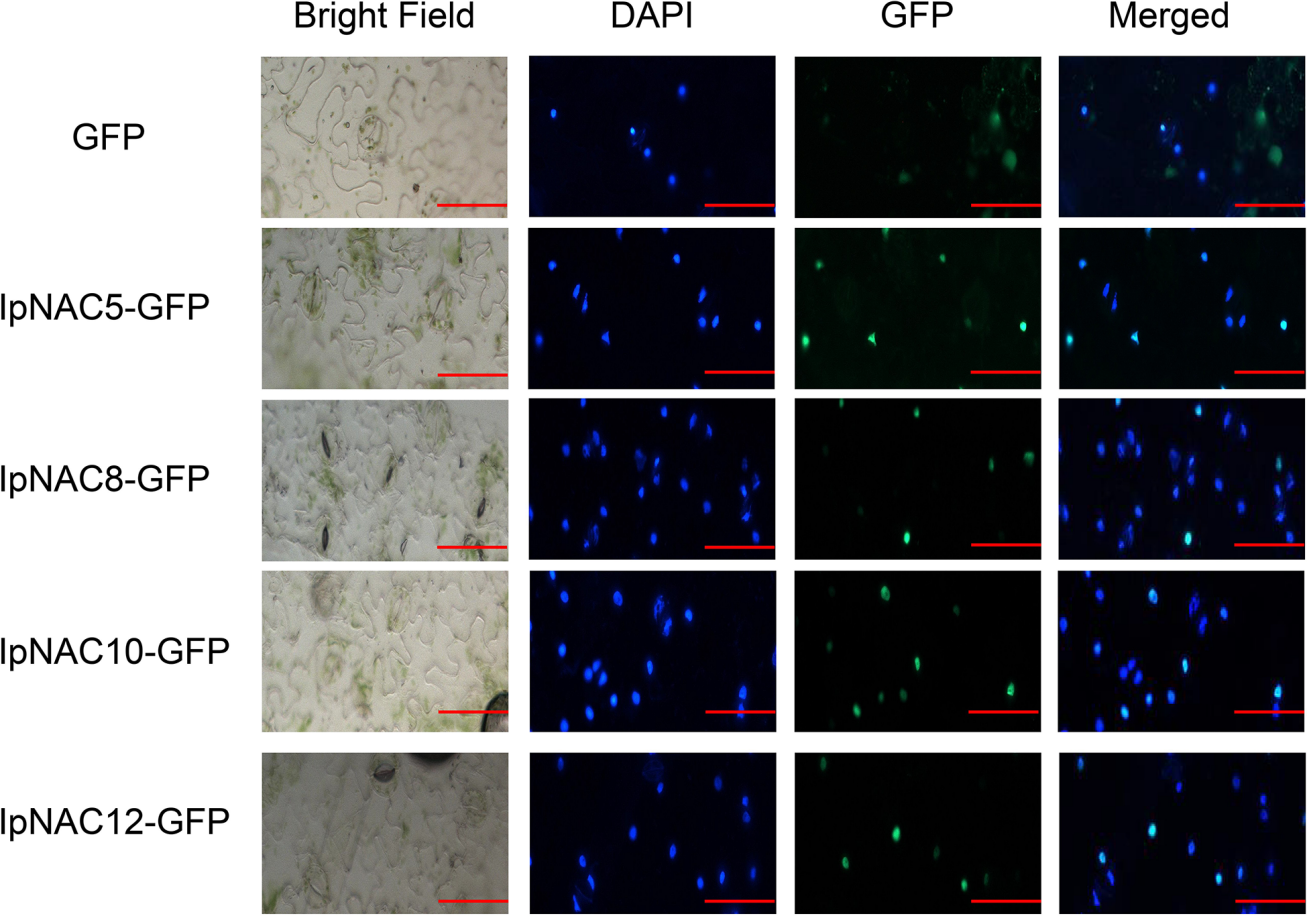
Subcellular localization of IpNAC5/8/10/12 in tobacco cells. IpNAC5/8/10/12-GFP and PHB-GFP were transiently expressed in tobacco. GFP fluorescence was observed 2 d after infection by 10 × 20-fold laser scanning. Photos were taken under bright and dark light for the detection of green fluorescent protein (GFP), diamino-2-phenylindole (DAPI), and binding (merging). Bars = 30 μm.

DNA- and TF-specific binding is involved in transcriptional regulation activities, and most TFs with substantial functions are located in the cell nucleus. We screened four candidate salt-tolerance genes and performed subcellular localization for IpNAC5/8/10/12 *in vivo* (**Figure 7**). The positions of IpNAC5/8/10/12 in plant cells were detected by GFP markers under the control of the CaMV 35S promoter. In the transient expression of GFP-IpNAC5/IpNAC8/IpNAC10/IpNAC12 in tobacco mesophyll protoplasts, the GFP signal was located in the nucleus and mixed with nuclear marker dye DAPI (**Figure 7**). However, GFP was located in the nucleus of tobacco mesophyll cytoplasm when tobacco mesophyll protoplasts carrying GFP alone were used as controls. These results suggest that GFP-IpNAC5, GFP-IpNAC8, GFP-IpNAC10, and GFP-IpNAC12 are located in the nucleus (**Figure 7**), which is consistent with the results presented in **Table 1**.

**Table 1:** The information of screened salt stress-responsive *Ipomoea pes-caprae IpNAC genes* in this study.

Name	Gene IDa	Gene location	Gene Length (bp)	Length (amino acids)	MW (kDa)	Isoelectric point (pI)	Subcellular localization	Subject annotation	Subject ID
*IpNAC1*	evm.TU.chr_0.2780	49282875-49285709	2834	269	30.93	5.94	Nucleus	NAC90-like	XP_031122657.1
*IpNAC2*	evm.TU.chr_12.1900	38917645- 38918836	1191	242	27.58	8.47	Nucleus	NAC1	AQZ36520.1
*IpNAC3*	evm.TU.chr_13.2033	41182054- 41184312	2258	261	29.35	8.29	Nucleus	NAC90-like	XP_019155111.1
*IpNAC4*	evm.TU.chr_13.2342	44337647- 44339364	1717	330	36.92	6.01	Nucleus	NAC87-like	XP_031126261.1
*IpNAC5*	evm.TU.chr_13.3468	52859128- 52861073	1945	339	38.22	6.54	Nucleus	NAC35-like	XP_019165208.1
*IpNAC6*	evm.TU.chr_14.1132	8670217 - 8671417	1200	338	37.79	8.52	Nucleus	NAC-JA2L-like	XP_031105753.1
*IpNAC7*	evm.TU.chr_14.925	6747525- 6749005	1480	251	28.16	6.39	Nucleus	NAC90-like	XP_031105272.1
*IpNAC8*	evm.TU.chr_3.63	316246- 317956	1710	295	34.23	6.54	Nucleus	NAC2-like	XP_019200440.1
*IpNAC9*	evm.TU.chr_5.3656	67821086- 67822648	1562	301	34.07	5.24	Nucleus	NAC92-like	XP_019185498.1
*IpNAC10*	evm.TU.chr_7.235	39755974- 39764569	8595	325	35.85	6.61	Nucleus	NAC72-like	XP_019183034.1
*IpNAC11*	evm.TU.chr_7.3373	61134772- 61138708	3936	344	39.21	6.33	Nucleus	NAC30-like	XP_031115981.1
*IpNAC12*	evm.TU.chr_7.684	4951286- 4952714	1428	283	32.1	6.39	Nucleus	NAC32-like	XP_031117584.1

MW, molecular weight; pI, isoelectric point.

### *IpNAC5/8/10/12* improved salt tolerance in transgenic *Arabidopsis*

**Figure 8:**
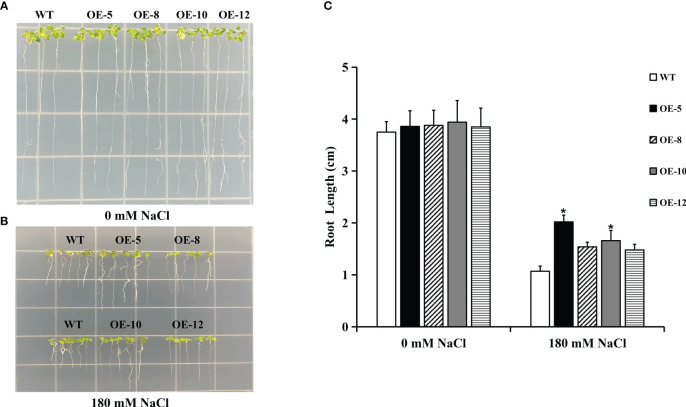
Transgenic plants overexpressing *IpNAC5/8/10/12* had improved root length under salt stress. **(A-C)** Comparisons of the root length between wild type (WT) and transgenic *Arabidopsis thaliana* plants grown on 1/2 MS + 180 mM NaCl for 10 d. Data are the means ± SE of three independent biological experiments. OE-5 is an *IpNAC5*-overexpressing plant, OE-8 is an *IpNAC8*-overexpressing plant, OE-10 is an *IpNAC10*-overexpressing plant, and OE-12 is an *IpNAC12*-overexpressing plant. The *p-values* were evaluated using Student’s *t*-test. Asterisks indicate the level of significance, The meaning of the symbol “*” is the level of significance, *0.01 < p < 0.05, between the WT and transgenic plants.

Heterologous overexpression in *Arabidopsis* is a useful technique for verifying gene function. The physiological phenotypes of the WT and transgenic *Arabidopsis* on 1/2 MS were similar (**Figure 8A**). At 180 mM NaCl, the root length of transgenic plants was longer than that of WT plants (**Figures 8B, C**). *Arabidopsis* overexpressing *IpNAC5/8/10/12* was highly salt tolerant at the post-germination stages. Among these, plants overexpressing *IpNAC5* and *IpNAC10* showed excellent characteristics.

### Na*^+^* homeostasis in *IpNAC5/8/10/12*-overexpressing SweetPotato roots

**Figure 9:**
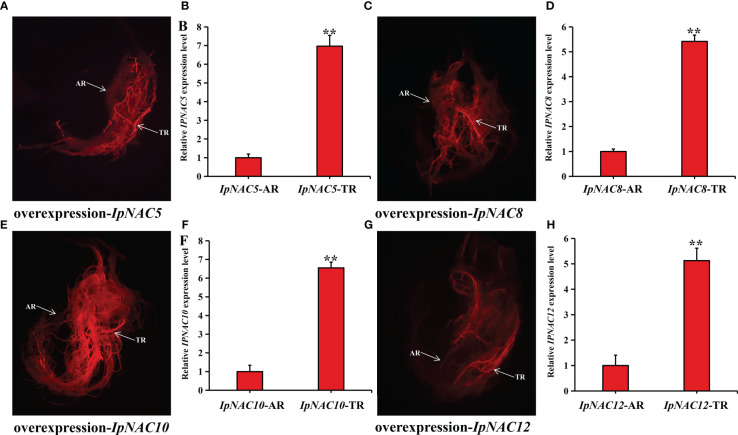
Preparation of *IpNAC5/8/10/12* transgenic sweetpotato roots. **(A, C, E, G)** DsRed fluorescence diagram of transgenic sweetpotato root. TR: positive transgenic roots; AR: non-transgenic roots. **(B, D, F, H)** Relative expression levels of *IpNAC5/8/10/12* in TR and AR. Asterisks indicate the level of significance, **p < 0.01. Gene levels of AR were used as a reference and set as 1.

**Figure 10:**
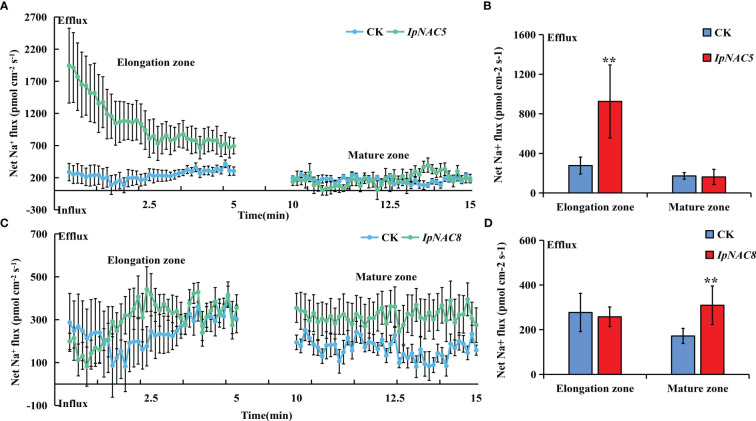
Na^+^ flux determination in *IpNAC5/8*-overexpressing sweetpotato roots. **(A, C)** The steady-state Na^+^ fluxes in the AR and TR, including the elongation and mature zones, after 24 h of NaCl treatment (150 mM). The standard errors of the means are represented by the bars. **(B, D)** Mean net Na^+^ efflux rates in **(A, C)**, respectively. Asterisks indicate the level of significance between the AR and TR groups at **p < 0.01.

**Figure 11:**
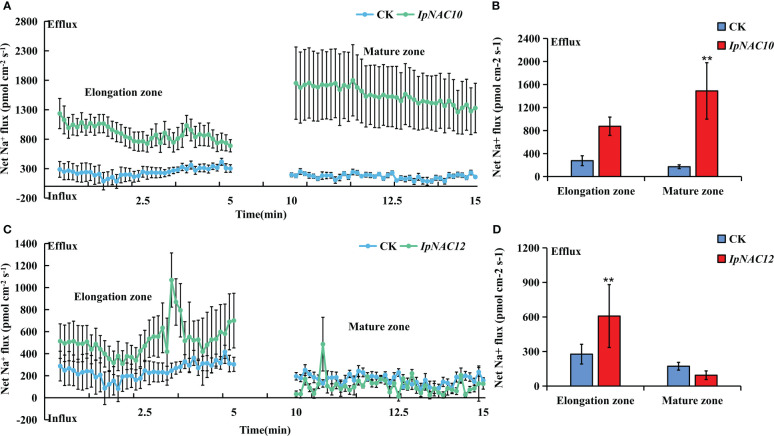
Na^+^ flux determination in *IpNAC10/12*-overexpressing sweetpotato roots. **(A, C)** The steady-state Na^+^ fluxes in the AR and TR, including the elongation and mature zones, after 24 h of NaCl treatment (150 mM). The standard errors of the means are represented by the bars. **(B, D)** Mean net Na^+^ efflux rates in **(A, C)**, respectively. Asterisks indicate the level of significance between the AR and TR groups at **p < 0.01.

To determine whether *IpNAC5/8/10/12* affect root Na^+^ homeostasis, we carried out root transgenics and obtained many complete transgenic roots (**Figure 9**). The target genes showed higher expression in the positive transgenic roots (TR) than in the non-transgenic roots (AR) (**Figures 9B, D, F, H**). The salt-induced Na*^+^* fluxes in the elongation and maturation zones of AR and TR were measured using the noninvasive microtest technique (NMT) after 24 h of NaCl stress (150 mM). Na*^+^* efflux in the elongation and mature regions of AR and TR differed (**Figure 10**, **Figure 11**). Among the four genes, the Na*^+^* efflux of the *IpNAC10*-overexpressing plant TR was more obvious than that of AR. The average Na*^+^* efflux rates in the two root zones of TR were 3.2- and 6.7-fold that of AR, respectively (**Figures 11A, B**). Interestingly, the Na*^+^* efflux of TR in the *IpNAC5*-overexpressing plant was 3.3-fold that of AR in the elongation zone, but there was no significant difference in the mature zone (**Figures 10A, B**). The average Na*^+^* efflux rate of TR in *IpNAC8* was 1.8-fold that of AR in the mature zone (**Figures 10C, D**). The average Na*^+^* efflux rate of TR in *IpNAC12* was 2.2-fold that of AR in the elongation zone (**Figures 11C, D**). These results indicate that *IpNAC5/8/10/12* overexpression probably activated the ion channel and increased the root Na^+^ ion efflux efficiency.

## Discussion

The influence of extreme weather is aggravating. Enhancing crop tolerance to respond to various abiotic stressors is essential for improving agricultural production efficiency. *Ipomoea pes-caprae*, a wild relative of sweetpotato, has significant biological characteristics in saline–alkali tolerance (Liu et al., 2020) and resistance to high temperature, drought (Suarez, 2011), and other abiotic stressors (Zhang et al., 2018). Compared with sweetpotato, *I*. *pes-caprae* has a smaller genome and is an excellent model for studying the function of sweetpotato genes. NAC proteins are a vital part of the signal transduction network and play an important regulatory role in plant responses to various stressors (Meng et al., 2022). However, NAC TF-related research reports mainly focus on model plants, and there are few reports on *I*. *pes-caprae*. Here, 12 salt-responsive *IpNAC* genes were isolated. The number of *IpNAC* genes was less than that of *ANAC* genes (**Table 1**). One reason may be that the genome of *I*. *pes-caprae* is small, and there were no large-scale repetitive events in the early stages of plant evolution. In *Arabidopsis*, a model plant, functional verification is clear (Ooka et al., 2003). Based on the classification of the phylogenetic tree of IpNAC and ANAC proteins, IpNACs were divided into four subfamilies (**Figure 2A**). IpNAC4 and IpNAC9 proteins may belong to the NAM subfamily. NAM are involved in flower formation (Hendelman et al., 2013) and leaf senescence (Wang et al., 2022). IpNAC2, IpNAC6, IpNAC8, IpNAC10, and IpNAC12 were assigned to ATAF (**Figure 2A**). The ATAF subfamily has many transcriptional activators or inhibitors that are crucial in plant stress resistance and hormone synthesis (Peng and Neff, 2021), indicating that IpNAC2, IpNAC8, and IpNAC12 may have similar roles. Among them, the IpNAC2 protein is NAC1, and its function is unknown. Based on other plants, NAC1 significantly contributes to increasing resistance to stress (You et al., 2014). The OsNAC7 subfamily contains only IpNAC11, which plays a major role in the response to abiotic stress and the regulation of plant growth (Wang et al., 2021). The ONAC22 subfamily significantly reduces the water loss rate and the transpiration rate to reduce drought stress (Peng et al., 2022). In addition, this subfamily improves salt tolerance by adjusting root length (Hong et al., 2016). As shown in **Figure 8**, the *IpNAC5* transgenic roots were relatively long. Collinearity analysis can describe the positional relationships of genes, as well as evolutionary and functional connections on chromosomes. Six *IpNAC* genes had a collinear relationship with *ANACs* (**Figure 2B**). Among them, *IpNAC6* had a collinear relationship with three *ANAC* genes (*AT1G52880*, *AT3G15500*, *AT4G27410*) and had many important regulatory functions. In addition, *AT4G27410* had a collinear relationship with *IpNAC6* and *IpNAC10*. *At4G27410* encodes a dehydration-induced NAC transcription factor and acts as a transcriptional activator in the ABA-mediated dehydration response (Fujita et al., 2004). *IpNAC6* and *IpNAC10* may enable stress resistance through ABA hormone regulation. **Figure 2** shows that most of the *IpNAC* genes have a close relationship with *ANAC* genes, indicating that their functions may be similar. The structure of the gene and the number of amino acid motifs play important roles. The 12 IpNAC proteins contained five NAC-specific motifs (A–E), indicating that IpNACs are highly conserved (**Figure 1**). Through the multiple sequence alignment of IpNAC proteins, the B and E domains had obvious differences, which may be an important reason for gene function diversification (**Supplementary Figure S1**). The UTR and intron–exon constitute the gene structure and, ultimately, through biological rules, perform biological functions. *IpNAC2, IpNAC6*, and *IpNAC7* lack an UTR, which indicates that the response expression may be slow (Srivastava et al., 2018) (**Figure 1**). Cis-acting elements are important molecular switches that work together with stress-inducible regulatory factors (Chanwala et al., 2022). The *IpNAC* promoter sequence contained some abiotic stress response elements and many hormone-responsive elements (**Figure 3**). This indicates that plant hormones play a central role in the regulation of the abiotic stress response. Different stress factors directly or indirectly affect gene expression levels. According to the gene expression level, we inferred that most of the *IpNAC* genes responded to induction under different stress treatments. Compared with different salt solutions, under the 300 mM NaCl solution, the treated *IpNACs* had the strongest ability to respond to the induction, followed by the 600 mM NaCl solution (**Figure 4**). With the increase in salinity, the time of the maximum gene expression level was delayed. The *IpNAC* gene expression level after 900 mM NaCl treatment was generally near the baseline or even downregulated. We speculate that the salt concentration may be too high, and that the plant reduces most of the biological reactions to maintain cell survival. In particular, *IpNAC10* showed the highest induction level under different salt solutions. Moreover, *IpNAC10* was the strongest inducer of the ABA response (about 500-fold) among *IpNACs* (**Figure 6**) and was also highly responsive to drought stress (about 150-fold) (**Figure 5**). This finding is similar to the results of previous study (Fujita et al., 2004). Plant root cells can absorb water only when their water potential is lower than the surrounding medium water potential (Erlandsson, 1979). We speculated that, because of the high salt solution content, the osmotic potential increases, which makes it difficult for plants to absorb water, leading to the high expression of *IpNAC10*, and its subsequent functions need further research. The characteristics of IpNAC8 and IpNAC12 were consistent with ATAF (**Figure 2**). Wu et al. (2009) found that *ATAF1* was significantly induced by high salinity and ABA, which enhanced the drought resistance of plants. Whether the function of the *IpNAC8* and *IpNAC12* genes is similar to that of *ATAF1* needs investigation. *IpNAC8* and *IpNAC12* were significantly higher under salt stress (about 25-fold and 300-fold, respectively) (**Figure 4**). Among the 12 genes, *IpNAC12* was the most strongly induced after drought stress (~500-fold) and SA stress (~2000-fold), but the expression level under high temperature treatment showed low sensitivity (**Figure 5**). At the same time, *IpNAC8* significantly responded to ABA (~200-fold) and MeJA stress (~150-fold). We speculate that *IpNAC10* and *IpNAC8* accomplish stress signal transduction not only through the ABA pathway but also through the SA and MeJA stress mechanisms. As a subtropical coastal plant, a high temperature is an essential element for *I*. *pes-caprae*. Under induction at 45 °C, *IpNAC5* had the highest expression (~500-fold). IpNAC5 shared a relatively high homology with ANAC036 (**Figure 2A**). Kato et al. (2010) suggested that *ANAC036* is induced by osmotic stress and salt stress. In terms of natural factors, salt and high temperature are inseparable. As a result, we speculated that high temperatures accelerated the evapotranspiration rate and increased the loss of water, leading the *IpNAC5* gene to be highly expressed in response to stress. Therefore, based on this analysis, we speculate that *IpNAC5/8/10/12* are important candidate genes for stress response. Further biological proof is necessary. Hence, we performed subcellular localization of IpNAC5, IpNAC8, IpNAC10 and IpNAC12 and found that they were located in the nucleus (**Figure 7**). Heterologous overexpression in *Arabidopsis* verified the gene function. We obtained transgenic seeds by overexpressing *IpNAC5/8/10/1*2 in *Arabidopsis*. T3 homozygous seeds were selected for the salt tolerance test. There was no significant difference between transgenic plants and WT plants in the no-salt medium (**Figure 8A**). Upon 180 mM NaCl stress, the root length and plant growth status of transgenic plants were greater than those of WT plants (**Figure 8B**). Compared with other *IpNAC5/8/10/12* genes, *IpNAC5* and *IpNAC10* had the strongest salt tolerance in the post-germination stages of *Arabidopsis*. Cellular Na*^+^* homeostasis and metabolic processes significantly affect plant salt tolerance (Zhang et al., 2017). By measuring Na*^+^* homeostasis in the transgenic sweetpotato roots, we found that the TR of *IpNAC10* had obvious Na*^+^* efflux in the mature and elongation zones compared with AR (**Figure 11A**). Interestingly, TR of *IpNAC5* and *IpNAC12* showed 3.3- and 2.2-fold more Na^+^ efflux in the elongation zone than AR, while there was no significant difference in the maturation zone (**Figure 10A**, **Figure 11C**). Similarly, the TR of *IpNAC8* showed significant Na^+^ efflux in the mature region compared to AR (**Figure 10C**). These results indicate that *IpNAC5/8/10/12* are highly responsive and protective in sweetpotato, resisting salt stress through Na^+^ ion channels, and were activated, leading to Na^+^ efflux in different regions of the sweetpotato roots.

Using *I*. *pes-caprae* as a basis, we utilized biotechnology to explore salt tolerance genes to provide relevant genetic resources and strategies for the genetic improvement of sweetpotato. At the same time, it is important to analyze the molecular regulatory mechanism of salt tolerance in *I*. *pes-caprae* to further plant adaptation to extreme climates.

## Conclusion

In this study, by combining transcriptome data with bioinformatics analysis, we identified 12 *IpNAC* genes in *I*. *pes-caprae*. Evolutionary collinearity, gene structure, and promoter cis-acting elements of *IpNACs* were analyzed. Different responses under a variety of abiotic and hormone stressors indicate that *IpNAC* genes are controlled by a variety of regulatory mechanisms. *IpNAC5/8/10/12* were screened as candidate genes. IpNAC5, IpNAC8, IpNAC10 and IpNAC12 localized in the nucleus. In addition, the root length and growth status of transgenic *Arabidopsis* under salt stress and the Na^+^ ion content in sweetpotato roots showed that *IpNAC5, IpNAC8, IpNAC10* and *IpNAC12* significantly enhanced plant salt tolerance. This study lays the foundation for further studies on the function of *IpNACs* under stress in *I*. *pes-caprae*. The candidate genes may contribute to the functional characterization of salt tolerance genes for breeding abiotic stress-resistant varieties of sweetpotato or other species. Taken together, these results provide new insights into the molecular response of *I*. *pes-caprae* and sweetpotato to salt stress.

## Data availability statement

The original contributions presented in the study are included in the article/**Supplementary Material**. Further inquiries can be directed to the corresponding author.

## Author contributions

QC planned and designed the research. YS, YL, and SX wrote the manuscript. YS, YuW, YD, LZ, YaW, DZ, XD, and ZZ conducted the research and analyzed the data. QC supervised the research and the manuscript. All authors read and approved the final manuscript. All authors contributed to the article and approved the submitted version.

## Footnotes

1
https://www.ncbi.nlm.nih.gov/orffinder/

2
https://web.expasy.org/protparam/

3
http://meme.nbcr.ne/meme/

4
http://gsds.cbi.pku.edu.cn/

5
https://www.arabidopsis.org/index.jsp

6
http://bioinformatics.psb.ugent.be/webtools/plantcare/html/

## Supplementary material

Supplementary Figure 1 IpNAC amino acid sequence alignment.

Supplementary Table 1 The information of screened salt stress-responsive Ipomoea pes-caprae IpNAC genes in this study.

Supplementary Table 2
*IpNAC* qRT-PCR primer design.
