# Supplementary figures and images for: Isolation, characterization, and functional verification of salt stress response genes of NAC transcription factors in Ipomoea pes-caprae
Source: Front Plant Sci. 2023 Feb 1;14:1119282. doi: 10.3389/fpls.2023.1119282 (PMC9929455; doi:10.3389/fpls.2023.1119282)

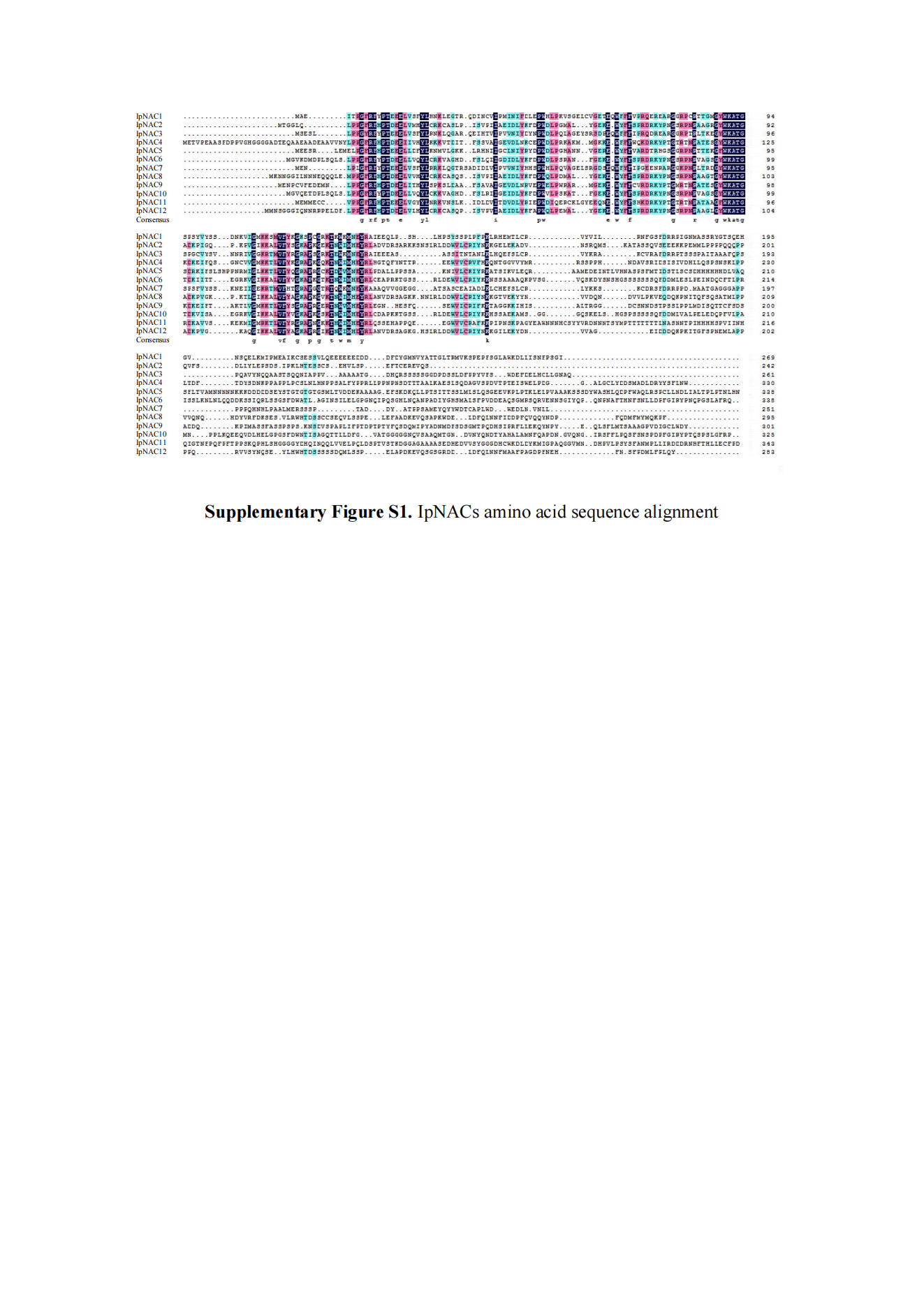

Supplement: Supplementary Figure 1 — IpNAC amino acid sequence alignment. [file Image_1.tif]
